# Vascularized bone regeneration accelerated by 3D-printed nanosilicate-functionalized polycaprolactone scaffold

**DOI:** 10.1093/rb/rbab061

**Published:** 2021-11-12

**Authors:** Xiongcheng Xu, Long Xiao, Yanmei Xu, Jin Zhuo, Xue Yang, Li Li, Nianqi Xiao, Jing Tao, Quan Zhong, Yanfen Li, Yuling Chen, Zhibin Du, Kai Luo

**Affiliations:** 1 Fujian Key Laboratory of Oral Diseases & Fujian Provincial Engineering Research Center of Oral Biomaterial & Stomatological Key Laboratory of Fujian College and University, School and Hospital of Stomatology, Fujian Medical University, Fuzhou 350002, China; 2 Institute of Stomatology & Laboratory of Oral Tissue Engineering, School and Hospital of Stomatology, Fujian Medical University, Fuzhou 350002, China; 3 Nanjing Stomatological Hospital, Medical School of Nanjing University, Nanjing 210008, China; 4 School of Mechanical, Medical, and Process Engineering, Queensland University of Technology, Brisbane, QLD 4059, Australia

**Keywords:** nanosilicate, bone regeneration, osteogenesis, angiogenesis

## Abstract

Critical oral-maxillofacial bone defects, damaged by trauma and tumors, not only affect the physiological functions and mental health of patients but are also highly challenging to reconstruct. Personalized biomaterials customized by 3D printing technology have the potential to match oral-maxillofacial bone repair and regeneration requirements. Laponite (LAP) nanosilicates have been added to biomaterials to achieve biofunctional modification owing to their excellent biocompatibility and bioactivity. Herein, porous nanosilicate-functionalized polycaprolactone (PCL/LAP) was fabricated by 3D printing technology, and its bioactivities in bone regeneration were investigated *in vitro* and *in vivo*. *In vitro* experiments demonstrated that PCL/LAP exhibited good cytocompatibility and enhanced the viability of bone marrow mesenchymal stem cells (BMSCs). PCL/LAP functioned to stimulate osteogenic differentiation of BMSCs at the mRNA and protein levels and elevated angiogenic gene expression and cytokine secretion. Moreover, BMSCs cultured on PCL/LAP promoted the angiogenesis potential of endothelial cells by angiogenic cytokine secretion. Then, PCL/LAP scaffolds were implanted into the calvarial defect model. Toxicological safety of PCL/LAP was confirmed, and significant enhancement of vascularized bone formation was observed. Taken together, 3D-printed PCL/LAP scaffolds with brilliant osteogenesis to enhance bone regeneration could be envisaged as an outstanding bone substitute for a promising change in oral-maxillofacial bone defect reconstruction.

## Introduction

Oral-maxillofacial bone tissue not only maintains the maxillofacial structure and appearance but also supports the functions of the oral maxillofacial system [1]. Critical oral-maxillofacial bone defects, such as damage by trauma and tumors, affect the physiological functions and mental health of patients [2]. Autograft bone harvested from the patient’s own tissue to transplant into the oral-maxillofacial bone defect remains an ideal procedure for bone reconstruction [3]. However, it creates a second defect site for autograft bone harvest and increases the risk of comorbidities due to the surgical procedure.

Growing developments in synthetic biomaterials show promise for replacing autografts in oral-maxillofacial bone tissue reconstruction applications [1, 4, 5]. With the evolution of personalized medicine, synthetic biomaterials have been increasingly used in the reconstruction of oral-maxillofacial bone defects through the advanced applications of cone-beam computed tomography combined with three-dimensional (3D) printing technology [6, 7]. Biomaterials customized by 3D printing technology for oral-maxillofacial bone defects could also save more time during surgery. 3D-printed biomaterials have the opportunity to replace conventional complicated procedures currently used for biomaterial fabrication [8]. The fused deposition modeling (FDM) technique, one of the most commonly used 3D printing technologies, could be used to fabricate a particular structure of scaffolds by ejecting the biomaterials layer by layer from a temperature-controlled nozzle [9]. FDM is flexible in feedstock and is cost-effective because it avoids the use of complex molds, extra dryness and cooling procedures. Polycaprolactone (PCL) is widely used in 3D printing technology for regenerative medicine due to its advantages of good biocompatibility, biodegradability and low immunogenicity [10, 11]. Its low melting temperature of ∼ 60°C makes it more processable, especially for scaffold fabrication *via* FDM. Moreover, PCL is degradable and only locally produces a minimal amount of acid during its hydrolysis and degradation [12]. 3D-printed PCL scaffolds have the potential to match the clinical requirements to facilitate oral-maxillofacial bone regeneration. However, PCL only maintains space during the regeneration process owing to a lack of pro-regenerative properties [13]. A variety of modifications have been applied to enrich the bioactivity of PCL, including surface modification or a combination of growth factors and bioactive inorganic nanoparticles [14, 15].

As a synthetic 2D nanosilicate, laponite (LAP) has shown great potential in tissue regeneration due to its multifaceted roles in regenerative medicine [16]. LAP can act as a filler in hydrogels to promote mechanical properties and tune bone regeneration by regulating the stiffness of hydrogels [16–18]. Furthermore, its hydrophilic surface and negative charge can stimulate protein adsorption to increase cell adhesion and can be used as a therapeutic delivery vehicle to deliver a range of molecules [18, 19]. An increasing number of studies have confirmed the bioactivity of LAP-containing biomaterials in the osteogenesis of BMSCs and osteoblasts and the promotion of bone regeneration in different bone defects [18, 20–22]. Most of them focused on incorporation of LAP into different polymers (such as PCL and PLGA) to obtain functionalized fibrous membranes *via* electrospinning for potential bone regeneration applications [22–26]. Gaharwar *et al.* demonstrated LAP-enriched electrospun PCL membranes support proliferation and osteogenic differentiation of BMSCs *in vitro* [22]. Liu *et al.* developed LAP-containing electrospun PLGA membranes to implant into rat periodontal defects and found that addition of LAP could facilitated periodontal bone regeneration *in vivo* [23]. However, electrospun membranes lack enough space-maintaining properties for large area bone regeneration. And the mechanism of LAP-containing PCL on bone regeneration is worth further exploring.

In the present study, we tested the hypothesis that a 3D-printed porous LAP-incorporated PCL (PCL/LAP) scaffold would accelerate osteogenesis of BMSCs and augment calvarial bone defect regeneration. Herein, PCL/LAP was prepared in the present study by a solvent-exchange method and was then fabricated by 3D printing technology to develop porous scaffolds. The effects of porous PCL/LAP scaffolds on cell viabilities, osteogenic differentiation and pro-angiogenic properties of BMSCs were systematically investigated. Moreover, a rat critical calvarial bone defect was used to explore the impacts of 3D-printed PCL/LAP scaffolds on biosafety and bone regeneration *in vivo*. This investigation offers an opportunity not only to illustrate the bioactivities of nanosilicate-incorporated PCL scaffold in bone regeneration *in vitro* and *in vivo*, but also to elucidate the effects of this scaffold on bone regeneration. This study suggests that nanosilicate-incorporated PCL could serve as a promising scaffold applied in oral-maxillofacial bone reconstruction.

## Materials and methods

### Preparation and characterization of samples

PCL/LAP composites were fabricated by a solvent-exchange method as previously reported [27]. PCL (Sigma-Aldrich, USA) with an average molecular weight of 80 000 g/mol was dissolved in dimethylacetamide (DMAc) (Sigma-Aldrich, USA) at 60°C to obtain a 5 wt% solution (Solution A). LAPONITE^®^ XLG was sourced from Nanocor Inc. (Beijing, China). LAPONITE^®^ XLG was dispersed in sterile ddH_2_O (Solution B), and DMAc was added to Solution B under sonication for 30 min to obtain a solution with a 5 μg/ml LAP concentration (Solution C). Solution C was mixed with Solution A under ultrasonication at 60°C for 6 h to produce final solutions with 5 wt% LAP. The final solutions were frozen at −20°C to form crystals. The supernatant was removed, and crystals were frozen at −80°C and lyophilized for ∼ 48 h to obtain composites. PCL powders treated with a similar procedure were set as controls.

The PCL and PCL/LAP scaffolds were fabricated by a 3D Bioplotter (Envisiontec, Germany). As recommended in the literature [12], the nozzle size and strand distance were 300 and 500 µm, respectively. Then, scaffolds with a diameter of 6.0 mm and a height of 1.0 mm were sterilized by ethylene oxide. Surfaces of PCL and PCL/LAP were imaged using scanning electron microscopy (SEM) (Hitachi S4800 FEG, Tokyo, Japan) and their elements were analyzed by energy dispersive spectrometry (EDX) (Hitachi S4800 FEG, Tokyo, Japan).

### BMSC culture and identification

All study protocols and animal care procedures were approved by the Animal Care and Use Committee of Fujian Medical University. Fifteen 4-week-old Sprague-Dawley rats were obtained from the animal resource center (SLAC Laboratory Animal Co, Ltd., Shanghai, China). BMSCs were isolated and cultured from rat femurs as previously described [28]. Colony formation assays were conducted and stained with 0.1% crystal violet (Sigma-Aldrich, USA). The culture medium of BMSCs was changed to osteoinductive medium (Cyagen, China) or adipoinductive medium (Cyagen, China) to confirm their multiple differentiation abilities. Alkaline phosphatase (ALP) staining was conducted using a BCIP/NBT Alkaline Phosphatase Color Development kit (Beyotime, China). Mineralization nodules were stained with Alizarin Red S (Sigma-Aldrich, USA). Lipid droplets of BMSCs were stained with Oil Red O (Sigma-Aldrich, USA). The surface markers of BMSCs were evaluated by flow cytometry. BMSCs were suspended with PBS-containing fluorescein isothiocyanate (FITC)-coupled antibodies against CD90 and CD105 (Abcam, USA). FITC-coupled nonspecific IgG (Abcam, USA) was used as an isotype control.

For the cell scaffold co-culture experiments, all scaffolds were sterilized by ethylene oxide. BMSCs were cultured on the samples placed in 24-well culture plates at an initial density of ∼ 2 × 10^4^ cells per well.

### Cell morphology and immunofluorescence staining

The cell/scaffold construct was fixed, permeabilized and blocked for immunofluorescence staining. The actin cytoskeleton was stained with Acti-stain 488 phalloidin (1:200; Cytoskeleton, USA). The cell nuclei were stained with 4′,6-diamidino-2-phenylindole (DAPI) solution (Beyotime, China). The stained cells were imaged using fluorescence microscopy (Olympus, Japan).

For RUNX2 protein evaluation, the primary antibody against RUNX2 (1:2000; Abcam, USA) was incubated for 2 h before Acti-stain 488 phalloidin and DAPI staining. An Alexa Fluor 647-conjugated secondary antibody (1:400, Abcam, USA) for RUNX2 was added for 1 h.

### Cell viability assay

A CCK-8 assay (Dojindo, Japan) was performed to measure cell viability by measuring the optical density value at 450 nm at different time points using an iMark microplate reader (iMark, Bio-Rad Laboratories, USA). The cell viabilities of BMSCs were calculated according to initial seeding number on each scaffold (Day 1).

### ALP and mineralization nodules analysis

ALP staining was conducted as described in the section ‘BMSC culture and identification’. To evaluate ALP activity, osteoblasts on samples were lysed with RIPA lysis buffer (Beyotime, China) and were assessed using an ALP assay kit (Jiancheng Inc, China) and a BCA protein assay kit (Beyotime, China) according to the manufacturer’s instructions. ALP activity was normalized to the total protein content of the cell lysate.

Alizarin red S staining was conducted as described in the section ‘BMSC culture and identification’. The stains were eluted with 10% cetylpyridinium chloride in 10 mM sodium phosphate (pH 7.0) for quantification analysis.

### Quantitative real-time PCR analysis

RNA was extracted from cells using TRIzol reagent (Life Technologies, USA) and transcribed using a PrimeScript RT reagent kit (Takara, Japan). Each gene expression level was determined using a LightCycler 480 real-time PCR system (Roche Diagnostics, Germany) with SYBR green PCR mix (Takara, Japan). The primers were synthesized by Sangon Biotech (Shanghai, China) and are shown in Table 1.

**Table 1.** Primer sequences used in quantitative real-time PCR

Gene	Primers
*GAPDH (rat)*	Forward: 5′-CGGCAAGTTCAACGGCACAGTCAAGG-3′
	Reverse: 5′-ACGACATACTCAGCACCAGCATCACC-3′
*ALP (rat)*	Forward: 5′-ACAAGGTGGTGGACGGTGAAC-3′
	Reverse: 5′-CGTGAAGCAGGTGAGCCATAGG-3′
*RUNX2 (rat)*	Forward: 5′-ACCAGCAGCACTCCATATCTCTAC-3′
	Reverse: 5′- CTTCCATCAGCGTCAACACCAT-3′
*Col-1α1 (rat)*	Forward: 5′-CAACAGACTGGCAACCTCAAGAAG-3′
	Reverse: 5′-CACAAGCGTGCTGTAGGTGAATC-3′
*VEGFA (rat)*	Forward: 5′-CGTCCTGTGTGCCCCTAAT-3′
	Reverse: 5′-TGGCTTTGGTGAGGTTTGAT-3′
*ANGPT-1 (rat)*	Forward: 5′-TATGGATGTGAATGAAGGAGGATGG-3′
	Reverse: 5′-ACTGCCTCTGACTGGTTATTGC-3′
*FGF-2 (rat)*	Forward: 5′-CTGGCTATGAAGGAAGATGGAC-3′
	Reverse: 5′-CGGTAAGTGTTGTAGTTATTGGAC-3′
*GAPDH (human)*	Forward: 5′-ACCCACTCCTCCACCTTTGAC-3′
	Reverse: 5′-TCCACCACCCTGTTGCTGTAG-3′
*HIF-1α (human)*	Forward: 5′-AGGACACAGATTTAGACTTGGAGATG-3′
	Reverse: 5′-CAGTGGTAGTGGTGGCATTAGC-3′
*eNOS(human)*	Forward: 5′-TTGTCTGCGGCGATGTTACC-3′
	Reverse: 5′-GCGTATGCGGCTTGTCACC-3′
*VEGFA (human)*	Forward: 5′-CGCTTACTCTCACCTGCTTCTG-3′
	Reverse: 5′-TCCAACAATGTGTCTCTTCTCTTCG-3′
*KDR (human)*	Forward: 5′-CGCAGAGTGAGGAAGGAGGAC-3′
	Reverse: 5′-CCGTAGGATGATGACAAGAAGTAGC-3′
*TEK (human)*	Forward: 5′-GCAGAGAACAACATAGGGTCAAGC-3′
	Reverse: 5′-AGGTCATTCCAGCAGAGCCAAG-3′
*FGFR1 (human)*	Forward: 5′-CTGGGAGAGGGCTGCTTTGG-3′
	Reverse: 5′-CACTTTGGTCACACGGTTGGG-3′

### Western blot

Proteins of BMSCs cultured on both scaffolds were extracted using RIPA lysis buffer. Proteins were fractionated by gel electrophoresis and transferred onto polyvinylidene difluoride membranes (Millipore, USA). The membranes were blocked with 5% nonfat milk and incubated with primary antibodies against RUNX2 (1:1000; Abcam, USA) and GAPDH (1:1000; Abcam, USA) at 4°C. Then, the membranes were washed with TBST and incubated with the secondary antibody (1:2000, Affinity, China). The protein levels were then assessed using enhanced chemiluminescence reagents.

### Enzyme-linked immunosorbent assay

The levels of VEGF-A, ANGPT-1 and FGF-2 (Abcam, USA) in supernatants of BMSCs cultured on both scaffolds were assessed using enzyme-linked immunosorbent assay (ELISA) kits. The supernatants were collected after 3 and 7 days of culturing and determined by ELISA as per the manufacturer’s instructions.

### Angiogenic properties analysis

BMSCs were cultured with PCL and PCL/LAP scaffolds, and the supernatants were collected at Day 7 and referred to as PCL-CM and PCL/LAP-CM, respectively. The human umbilical vein endothelial cells (HUVECs) line EA.hy 926 was acquired from the Cell Bank of the Chinese Academy of Sciences and was cultured in Dulbecco’s modified Eagle’s medium (DMEM) containing 10% FBS supplemented with the collected supernatants at a ratio of 1:1.

A tube formation assay was conducted as previously described [29]. Briefly, Matrigel matrix (Corning, USA) was added to a 96-well plate. HUVECs were seeded on top of the solidified matrix solution with the supernatants. The lengths of tube-like structures were measured using ImageJ software.

For angiogenesis-related gene expression analysis, HUVECs were treated with the supernatants for 3 days and were then assessed by real-time PCR, as described previously.

### Rat calvarial bone defect model and analysis

The procedures were authorized by the Animal Care and Use Committee of Fujian Medical University. All rats were anesthetized *via* intraperitoneal injection of 40 mg/kg ketamine. Critical-sized defects with a 6-mm diameter were prepared in 8-week-old Sprague-Dawley rat calvaria, and scaffolds were placed in the defects. All rats were sacrificed 12 weeks after implantation.

The fixed calvaria were scanned by a microcomputed tomography (micro-CT) scanner (SCANCO µCT50, Switzerland). Bone volume per total volume ratio (BV/TV), bone mineral density (BMD) and new bone formation rate calculations were performed using CTAn software (gray value > 1000).

The calvaria were decalcified in 10% ethylenediaminetetraacetic acid for ∼ 1 month and were then dehydrated and embedded. Consecutive sections were obtained from the defect area. H&E, Masson’s trichrome and Alizarin red S staining were conducted to assess new bone formation. The new bone formation areas were assessed using ImageJ software.

Immunohistochemistry was carried out following the manufacturer’s instructions. Briefly, specimens were immersed in antigen retrieval solution, blocked with 1% BSA and incubated with primary antibodies against rat CD31 (1:2000; Abcam, USA) as a vascular endothelial cell surface marker. Alexa Fluor 488-conjugated secondary antibody (1:400) was incubated, and nuclei were stained with DAPI. The CD31-positive stained area was assessed using ImageJ software.

### Statistical analysis

Data are expressed as the mean ± standard deviation. The t-test or one-factor analysis of variance followed by Tukey’s HSD *post hoc* test was used to evaluate the differences among groups. Significance was accepted at *P* < 0.05 for all tests.

## Results

### Preparation and characterization of 3D-printed PCL/LAP scaffolds

**Figure 1.**
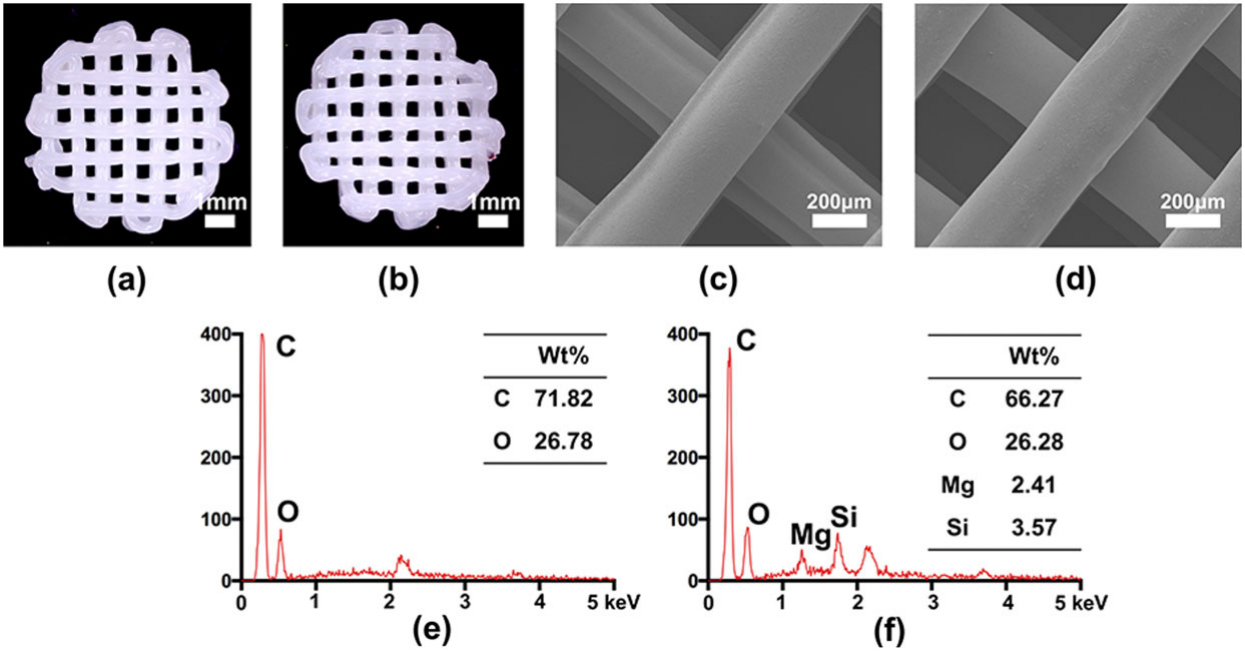
Preparation and characterization of 3D-printed PCL/LAP scaffolds. Micrographs of PCL (**a**) and PCL/LAP (**b**) scaffolds. SEM of PCL (**c**) and PCL/LAP (**d**) scaffolds. EDX of PCL (**e**) and PCL/LAP (**f**)

**Figure 2.**
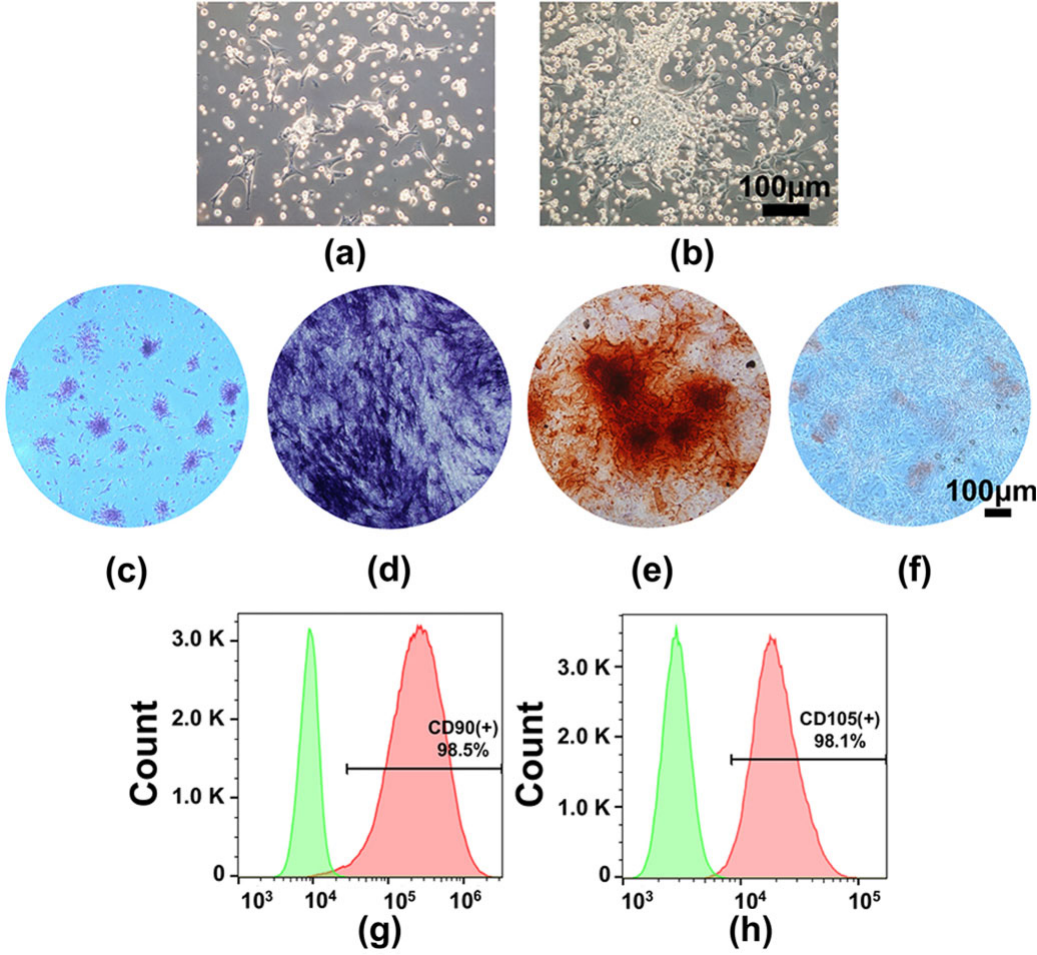
Culture and characterization of BMSCs. BMSCs isolated and cultured from rat femurs at Day 1 (**a**) and Day 5 (**b**); (**c**) colony-forming unit formation; (**d**) ALP staining; (**e**) Alizarin red S staining; (**f**) Oil Red S staining. Flow cytometric analysis of CD90 (**g**) and CD105 (**h**)

The 3D-printed porous PCL and PCL/LAP scaffolds are shown in Fig. 1a and b. Both porous scaffolds fabricated by 3D printing technology had microsized pores and regular shapes. The surfaces of 3D-printed PCL and PCL/LAP scaffolds were observed by SEM (Fig. 2c and d). Both scaffolds had relatively smooth surfaces and regular strands.

The elements of PCL and PCL/LAP were analyzed by EDX. As shown in Fig. 1e, only C and O were present in the PCL scaffolds. Both Mg and Si could be detected in PCL/LAP scaffolds by EDX patterns (Fig. 1f), which confirmed the presence of LAP in PCL/LAP.

### Culture and characterization of BMSCs

**Figure 3.**
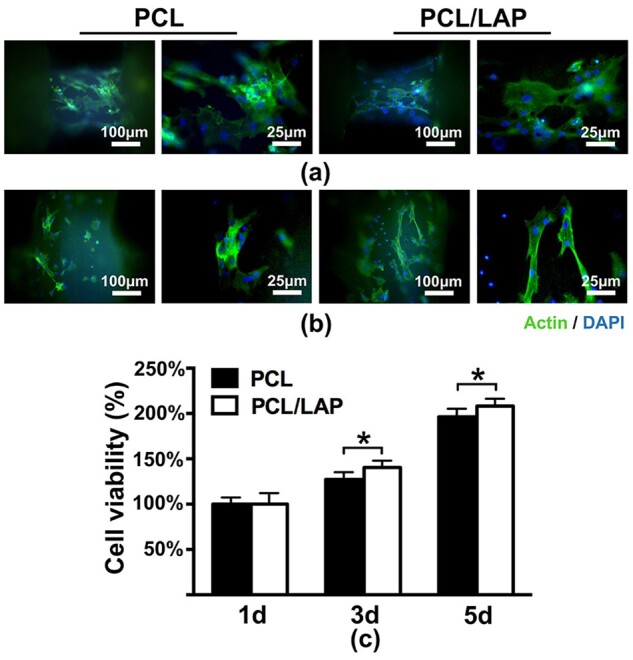
Morphologies and viabilities of BMSCs on 3D-printed PCL/LAP scaffolds. (**a**, **b**) Immunofluorescence staining of BMSCs on PCL and PCL/LAP scaffolds. (**c**) Proliferation of BMSCs on both scaffolds. *P < 0.05

As shown in Fig. 2a, BMSCs adhered to the culture plates with round and spindle-like morphology at Day 1 and proliferated readily to form colonies after 5 days of culture (Fig. 2b). Figure 2c indicates that BMSCs possessed good colony-forming ability. Multiple differentiation potentials were determined by ALP, Alizarin red S and Oil Red O staining (Fig. 2d and f), which confirmed the multiple differentiation abilities of BMSCs. Flow cytometric analysis indicated that BMSCs were positive for the mesenchymal stem cell-specific surface markers CD90 and CD105 (Fig. 3g and h). These results suggested that BMSCs could be successfully cultured from rat femurs.

### Morphologies and viabilities of BMSCs cultured on 3D-printed PCL/LAP scaffolds

Fluorescence microscopy was used to evaluate the morphologies of BMSCs cultured on 3D-printed porous PCL/LAP scaffolds. Figure 3a and b shows the cytoskeletons of BMSCs on different strands of porous PCL and PCL/LAP scaffolds. The addition of LAP to the PCL had no significant effect on the cell morphology of BMSCs. BMSCs on different stands showed spreading shapes.

As shown in Fig. 3c, the CCK-8 assay indicated that BMSCs cultured on both scaffolds exhibited similar increasing trends over 5 days of culturing. After 3 days of culture, BMSCs cultured on PCL/LAP were significantly enhanced compared with those cultured on PCL. These results demonstrated that 3D-printed porous PCL/LAP scaffolds exhibited good biocompatibility and excellent BMSC viabilities.

### 3D-printed PCL/LAP scaffolds enhanced the osteogenesis of BMSCs

**Figure 4.**
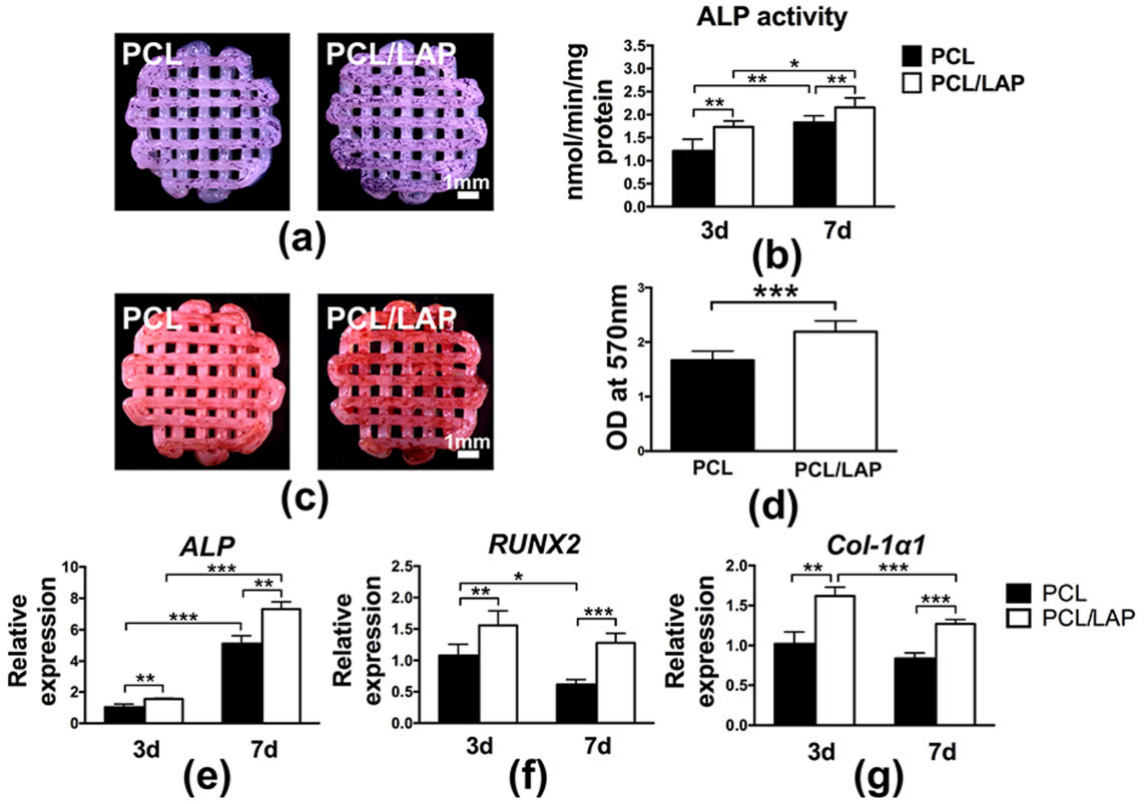
Osteogenesis of BMSCs on 3D-printed PCL/LAP scaffolds. ALP staining (**a**) and activity (**b**). Alizarin red S staining (**c**) and quantification (**d**). Expression of the osteogenic differentiation genes *ALP* (**e**), *RUNX2* (**f**) and *col-1α1* (**g**). *P < 0.05, **P < 0.01 and ***P < 0.001

The effects of 3D-printed PCL/LAP on the osteogenesis of BMSCs were determined by ALP staining and activity, mineralized nodule formation and osteogenic gene expression analysis. On Day 7, ALP staining was employed to assess the extent of osteogenic differentiation (Fig. 4a). Fewer ALP-positive cells were found on PCL scaffold. In contrast, higher intensity and darker staining were observed on PCL/LAP scaffolds. Then, an ALP activity assay was performed at Days 3 and 7 (Fig. 4b). BMSCs cultured on PCL/LAP scaffolds showed significantly higher levels of ALP at Days 3 and 7, which was similar to the ALP staining results.

Mineralized nodule formation was evaluated by Alizarin red S staining. As shown in Fig. 3c, mineralized nodules were expressed as red nodule-like staining. More red nodule-like stains were found on PCL/LAP scaffolds than on PCL scaffolds after 21 days of culturing. For quantitative analysis, Alizarin red S was extracted from the stained scaffolds. The results showed that PCL/LAP markedly promoted the mineralization of BMSCs (Fig. 4d).

To further assess the impacts of 3D-printed porous PCL/LAP scaffolds on the osteogenic differentiation of BMSCs, *ALP*, *RUNX2* and *Col-1α1* mRNA expression levels were evaluated. After 3 and 7 days of culturing, the mRNA expression levels of these osteogenic differentiation-related genes were significantly increased in PCL/LAP scaffolds in comparison to PCL scaffolds (Fig. 4e and f). *ALP* expression gradually increased from Days 3 to 7.

Based on the above results, 3D-printed porous PCL/LAP scaffolds synergistically enhanced the osteogenesis of BMSCs, as reflected by increased levels of ALP activity, mineralized matrix formation and osteogenic gene expression.

### 3D-printed PCL/LAP scaffolds increased RUNX2 protein expression in BMSCs

**Figure 5.**
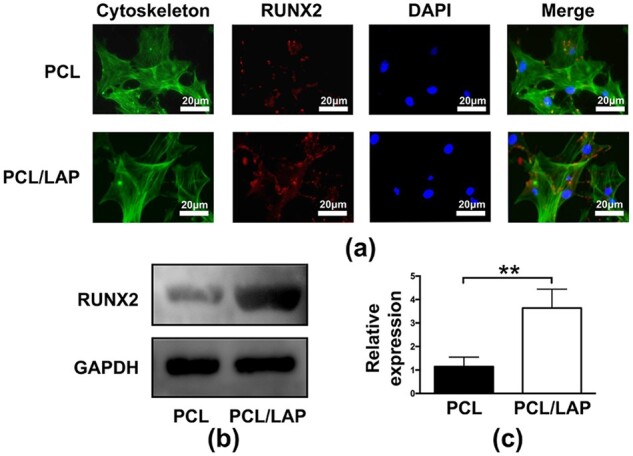
RUNX2 protein expression of BMSCs cultured on 3D-printed PCL/LAP scaffolds. (**a**) Immunofluorescent staining of RUNX2. (**b**, **c**) WB analysis of RUNX2. **P < 0.01

The expression of the RUNX2 protein in BMSCs cultured on 3D-printed porous PCL/LAP scaffolds was measured by immunofluorescence staining and Western blot (WB) analysis. As shown in Fig. 5a, obviously higher RUNX2 protein levels were detected in BMSCs on PCL/LAP scaffolds than in those on PCL scaffolds. Furthermore, WB evaluation results indicated that PCL/LAP dramatically promoted the expression of RUNX2 protein in BMSCs (Fig. 5b and c). These findings were in accordance with the PCR results. Collectively, these observations strongly suggest that 3D-printed PCL/LAP scaffolds enhance the osteogenesis of BMSCs *in vitro*.

### BMSCs cultured on 3D-printed PCL/LAP scaffolds promoted angiogenesis *in vitro*

**Figure 6.**
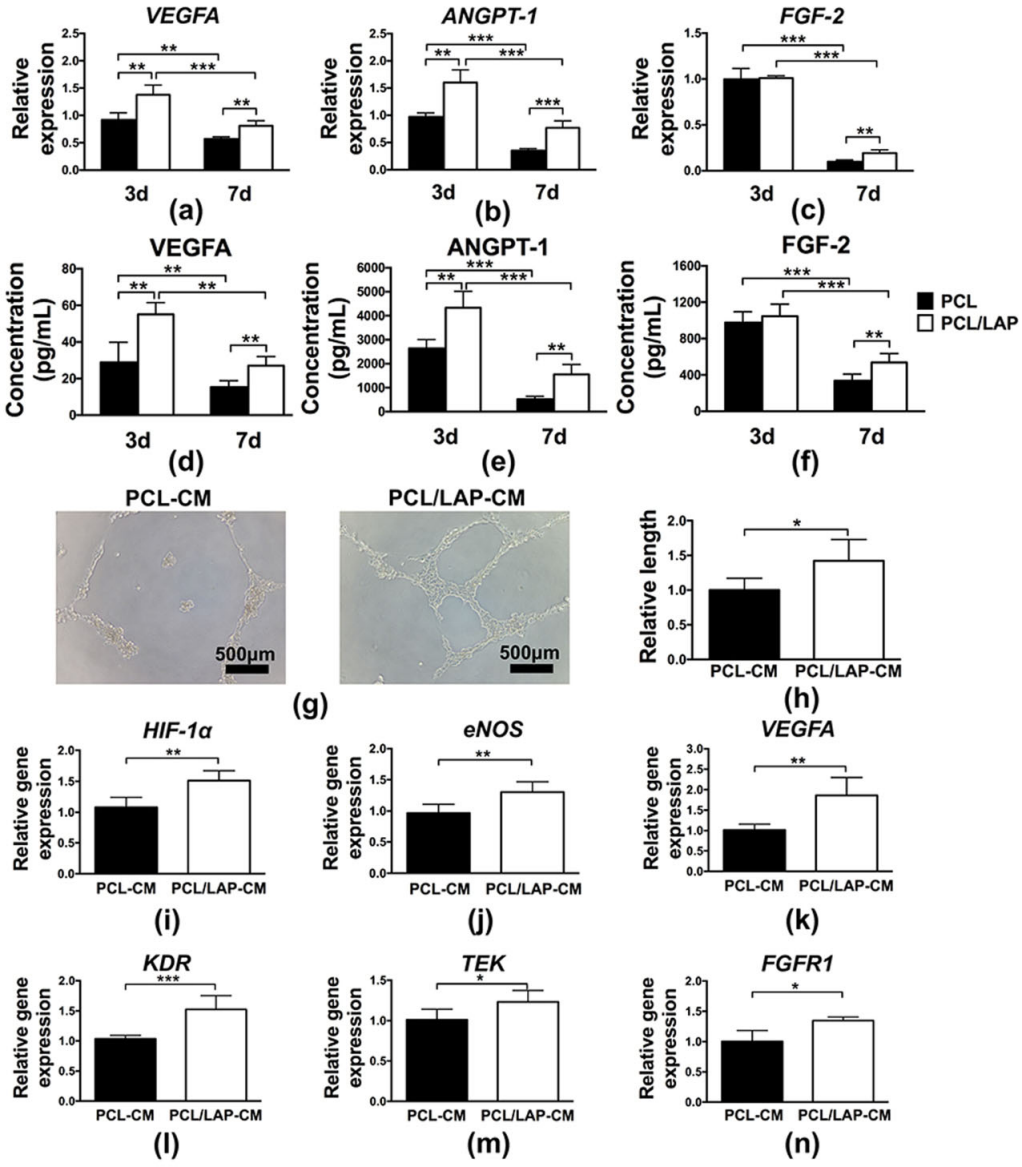
BMSCs cultured on 3D-printed PCL/LAP scaffolds promoted angiogenesis. Angiogenic cytokine (*VEGFA*, *ANGPT-1* and *FGF-2*) expression of BMSCs cultured on 3D-printed PCL/LAP scaffolds at gene (**a**–**c**) and protein (**d**–**f**) level. (**g**, **h**) Tube formation assays of HUVECs treated with supernatants of BMSCs cultured on PCL/LAP scaffolds. (**i**–**n**) Angiogenesis-related gene expression of HUVECs treated with supernatants of BMSCs cultured on PCL/LAP scaffolds. *P < 0.05, **P < 0.01 and ***P < 0.001

As shown in Fig. 6a–c, angiogenic cytokine expression in BMSCs cultured on 3D-printed PCL/LAP scaffolds was evaluated at the mRNA level. Obvious enhancements in angiogenic genes (*VEGFA, ANGPT-1* and *FGF-2*) were found in the PCL/LAP group. The expression of angiogenic proteins (VEGFA, ANGPT-1 and FGF-2) was also assessed using ELISA. Figure 6d–f indicated the protein levels of angiogenic cytokine were consistent with mRNA level. Angiogenic cytokines expressed in higher level at Day 3 compared with Day 7 in gene and protein levels.

Further experiments were performed to evaluate whether BMSC-cultured PCL/LAP would regulate angiogenesis in a paracrine manner. Figure 6g shows that HUVECs treated with PCL/LAP-CM group supernatants formed more tube-like structures than the PCL-CM group. The length of the tubes in the PCL/LAP-CM group was significantly longer than that formed by HUVECs in the PCL group (Fig. 6h). Then, angiogenesis-related gene expression was evaluated by quantitative real-time PCR analysis. HUVECs treated with PCL/LAP-CM group supernatants exhibited enhanced angiogenesis-related gene expression (*HIF-1α, eNOS* and *VEGFA*). The expression levels of angiogenic cytokine receptors (*KDR, TEK* and *FGFR1*) in HUVECs treated with PCL/LAP-CM group supernatants were also increased compared with those in HUVECs treated with PCL-CM group supernatants (Fig. 6i–n).

Taken together, the results demonstrated that HUVECs incubated with supernatants from BMSCs cultured on PCL/LAP facilitated tube-like structure formation and angiogenesis-related gene expression. These results suggest that BMSCs cultured on 3D-printed PCL/LAP scaffolds promoted the angiogenesis of HUVECs *in vitro*.

### Toxicological safety of 3D-printed PCL/LAP scaffolds *in vivo*

**Figure 7.**
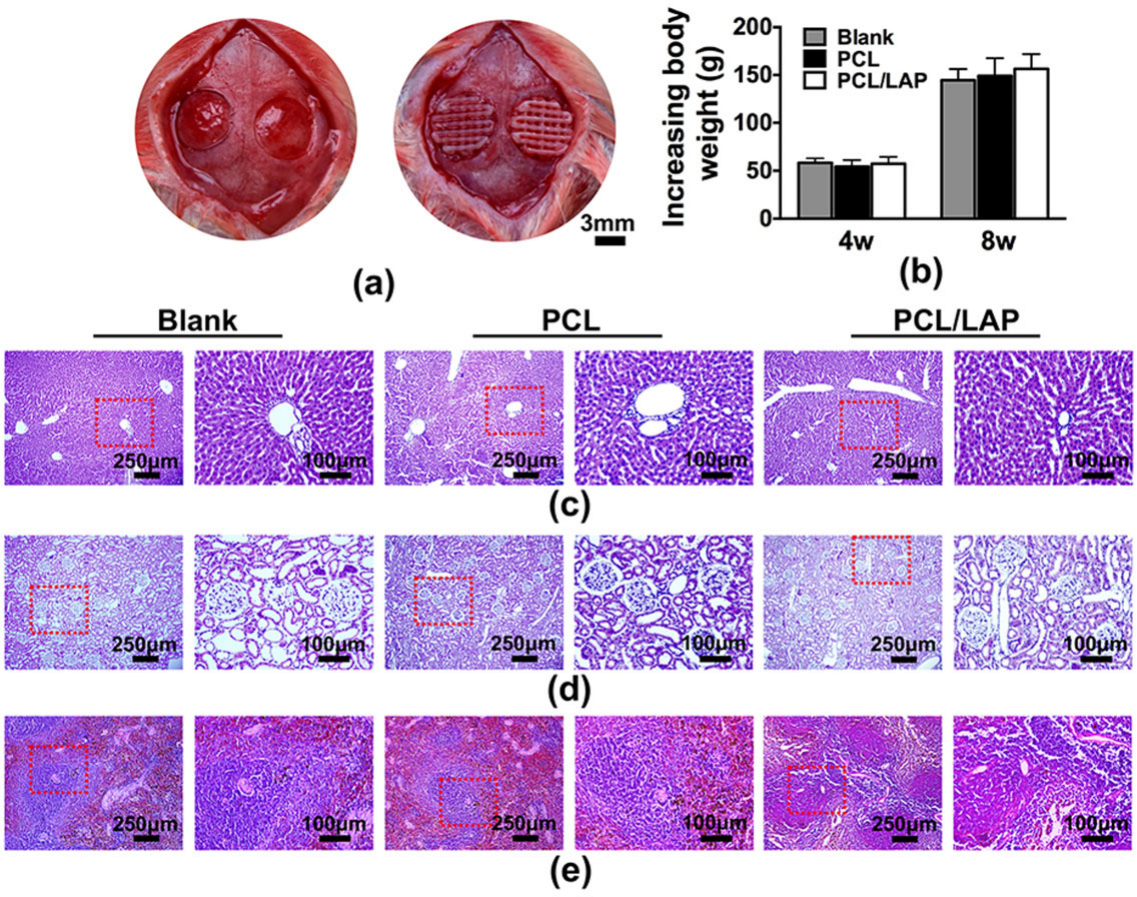
Toxicological safety of 3D-printed PCL/LAP scaffolds *in vivo*. (**a**) Surgical procedures for scaffold implantation. (**b**) Body weight changes of rats after implantation. H&E staining of the liver (**c**), kidney (**d**) and spleen (**e**) of rats after 3D-printed PCL/LAP scaffold implantation

Figure 7a shows the surgical procedures of 3D-printed scaffold implantation in rat calvarial bone defects. After 4 and 8 weeks of implantation, body weight changes of rats in the blank, PCL and PCL/LAP groups did not exhibit statistically significant differences (Fig. 7b). Furthermore, histological sections of the liver, kidney and spleen showed no evident toxicity in the blank, PCL and PCL/LAP groups (Fig. 7c–e). These findings indicated that 3D-printed porous PCL/LAP exhibited toxicological safety *in vivo*.

### 3D-printed PCL/LAP scaffolds augmented vascularized bone regeneration of calvarial defects *in vivo*

**Figure 8.**
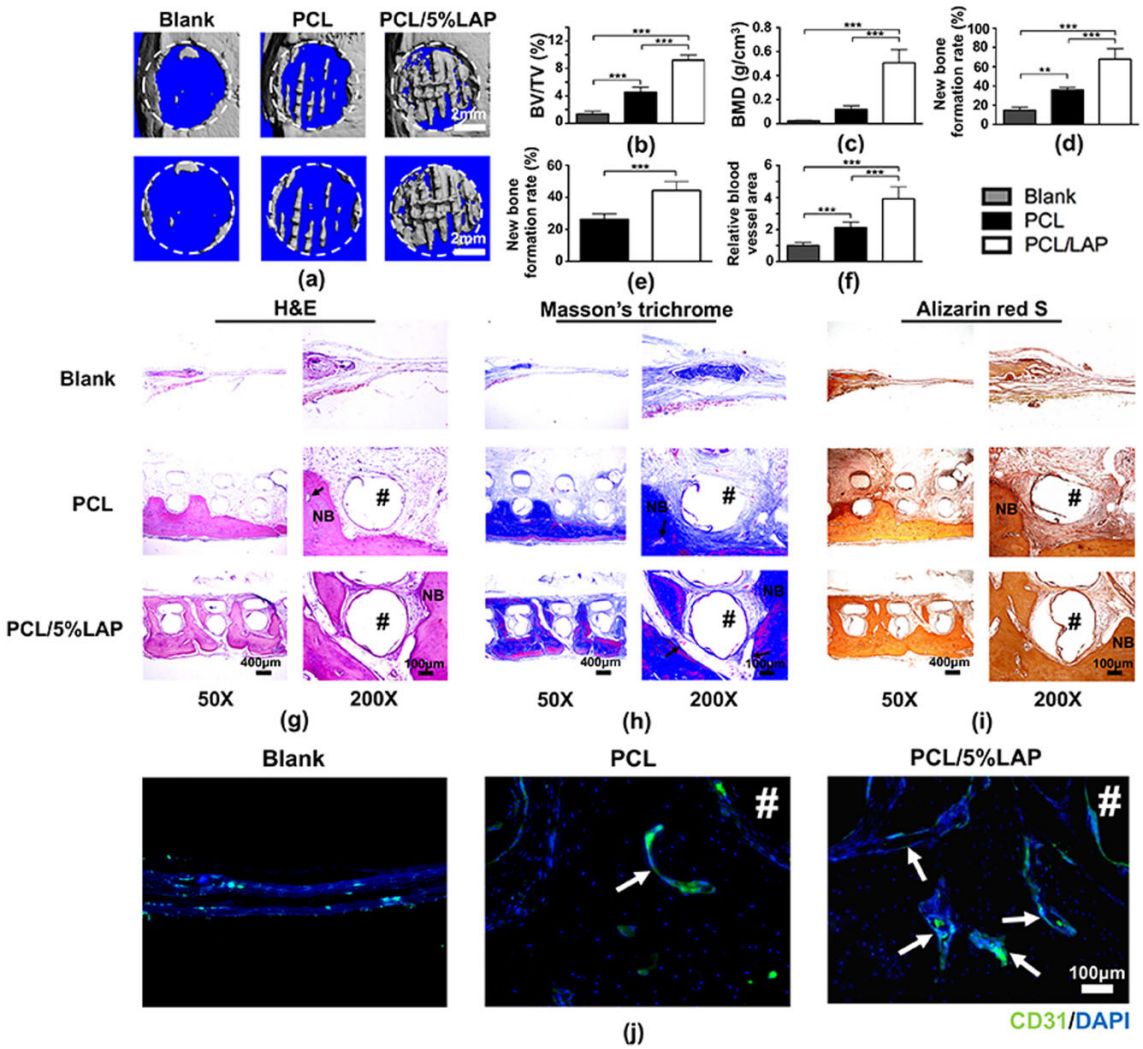
3D-printed PCL/LAP scaffolds accelerated rat calvarial bone defect regeneration *in vivo*. Representative micro-CT images (**a**) and the BV/TV (**b**), BMD (**c**) and new bone formation rate (**d**) of each group. (**e**) Percentages of new bone formation on the histological sections. (**f**) Quantitative analysis of blood vessel areas. Representative images of H&E (**g**), Masson’s trichrome (**h**), Alizarin red S (**i**) staining and immunohistochemical images of CD31-positive cells in defect areas. Arrows indicate blood vessels. Pound signs (#) indicate PCL or PCL/5%LAP scaffolds. NB, newly formed bone. **P < 0.01 and ***P < 0.001

Both PCL and PCL/LAP porous scaffolds were fabricated by 3D printing technology and implanted into rat calvarial defect models for 12 weeks. New bone formations in calvarial defects were analyzed by micro-CT to evaluate the role of PCL/LAP in bone regeneration *in vivo* (Fig. 8a). Twelve weeks after implantation, obvious new bone formation in the PCL/LAP group was detected in rat calvarial defect areas compared to those in the blank and PCL groups (Fig. 8a). The new bone formation rate of the PCL/LAP group was markedly higher than that of the PCL group by micro-CT analysis (Fig. 8d). The micro-CT parameters of BV/TV and BMD were also analyzed (Fig. 8b and c). Statistically enhanced levels of BV/TV and BMD were observed in the PCL/LAP group.

To further assess new bone formation after implantation, numerous histological analyses were conducted. More mature mineralized bone formation near the implanted scaffolds was observed in the PCL/LAP group than in the PCL group by H&E, Masson’s trichrome and Alizarin red S staining (Fig. 8g–i). Only a slight change could be observed in the blank group without implanted scaffolds. Quantitative data indicated that the new bone formation rate in the PCL/LAP group was significantly higher than that in the PCL group (Fig. 8e). These results were consistent with the micro-CT analyses. Moreover, new bone formation in the PCL group was predominantly located below the scaffold near the endocranium. However, mature, well-formed bone penetrated and filled the spaces in the scaffolds in the PCL/LAP group, which confirmed that 3D-printed PCL/LAP scaffolds possessed enhanced osteogenic properties.

To measure angiogenesis after implantation, CD31, a marker of blood vessel endothelial cells, was employed to explore new vessel formation in new bone areas by immunofluorescent staining (Fig. 8j). There were more CD31-positive cells in PCL/LAP scaffolds than in PCL scaffolds. Obviously increased areas of CD31-positive cells were exhibited in PCL/LAP scaffolds compared to PCL scaffolds. Quantitative analysis of the blood vessel areas was conducted. As shown in Fig. 8f, the blood vessel areas in PCL/LAP scaffolds were significantly higher than those in PCL scaffolds. These results indicated that 3D-printed porous PCL/LAP exhibited profound effects on blood vessel formation during bone regeneration in calvarial defects.

## Discussion

3D printing technology has made great progress in customizing biomaterials for bone tissue regeneration, which has great potential for replacing autografts as the primary choice in oral-maxillofacial bone defect reconstruction with customized scaffolds. Herein, a 3D-printed nanosilicate-incorporated PCL scaffold with excellent bioactivities was developed and evaluated to determine its potential utility in oral-maxillofacial bone defect reconstruction. The nanosilicate-incorporated PCL possessed brilliant biocompatibility and enhanced the proliferation and osteogenic differentiation of BMSCs *in vitro*. Moreover, BMSCs cultured on PCL/LAP promoted angiogenesis potential by angiogenic cytokine secretion. 3D-printed PCL/LAP scaffolds exerted good biosafety and pro-regenerative capacity after implantation into rat calvarial bone defects *in vivo*. These results provide a novel perspective for oral-maxillofacial bone defect reconstruction applications of 3D-printed nanosilicate-incorporated PCL.

3D-printed biomaterials have attracted considerable attention in bone tissue regeneration due to their flexible and convenient manipulation [8]. Scaffolds with porous structures ensure the transport of oxygen and nutrients, ingrowth of vasculature and migration of mesenchymal stem cells [30]. Previous studies have indicated that scaffolds with pore sizes between 300 and 500 μm and porosities higher than 50% could not only promote cell proliferation and osteogenic differentiation but also not jeopardize the mechanical properties of scaffolds [31–33]. The homogeneous cubic pore geometry of porous scaffolds could enhance the attachment and recruitment of mesenchymal cells [33–35]. Hence, these parameters were chosen in the current study to fabricate porous scaffolds *via* 3D printing technology. A series of nanosilicate-incorporated PCLs with different amounts of LAP particles were developed in our previous works, and we found that PCL with 5 wt% LAP showed excellent biological properties in rat osteoblasts. Therefore, an innovative porous scaffold with 5 wt% LAP incorporation was customized by 3D printing technology. To the best of our knowledge, this is the first study to describe the potential application of 3D-printed nanosilicate-incorporated PCL scaffolds in oral-maxillofacial bone tissue regeneration.

Intramembranous ossification is the main process of oral-maxillofacial bone regeneration, which recruits and enriches BMSCs to the regenerative site and then induces osteogenesis and angiogenesis after biomaterial implantation [1]. Attachment and recruitment of BMSCs occur on the surface of biomaterials after implantation. As shown in Fig. 3, BMSCs assumed a well-spread shape and proliferated more quickly on the PCL/LAP scaffold, which indicated that LAP incorporation positively stimulated the viability of BMSCs. These results were similar to previous studies focused on LAP-containing biomaterials [19, 22, 36, 37]. Some cellular and molecular evidence suggests that LAP nanosilicates possess brilliant osteoinductive properties to induce osteogenic differentiation of BMSCs in the absence of osteogenic medium [22, 37, 38]. In the current study, the 3D-printed PCL/LAP scaffold led to markedly higher ALP activity, and superior mineralized extracellular matrix formation also occurred on the porous PCL/LAP scaffold. Moreover, the PCL/LAP scaffold upregulated the expression of *RUNX2, ALP* and *Col-1α1* in BMSCs. RUNX2, a transcription factor involved in bone formation, activates bone-specific gene transcription promoters (including *ALP* and *Col-1α1*) during the early stage of osteogenic differentiation [39]. Therefore, it is reasonable to presume that the PCL/LAP scaffold stimulated extracellular matrix mineralization by accelerating Col-1α1 synthesis to provide a matrix for osteoid formation, which might be attributed to the increased RUNX2 level induced by LAP released from PCL/LAP. Similar trends were observed in previous studies of LAP-containing biomaterials [21, 22, 40, 41].

Vasculature acts as a communicative network in bone regeneration and supplies the regeneration site with oxygen, nutrients and growth factors as required. Angiogenesis plays a vital role in the bone regeneration process by metabolically activating new blood vessel invasion [42]. In the present study, 3D-printed porous PCL/LAP scaffolds increased the gene and protein expression of angiogenic cytokines (VEGFA, ANGPT-1 and FGF-2) in BMSCs, and the expression levels of their receptors (KDR, TEK and FGFR1) in endothelial cells were accordingly elevated after treatment with supernatants of BMSCs cultured on PCL/LAP scaffolds. Furthermore, more tube-like structure formation and increased angiogenesis-related gene expression were observed in the present study. Therefore, we speculated that BMSCs cultured on PCL/LAP scaffolds promoted the angiogenesis of endothelial cells in a paracrine manner. In addition, LAP released from PCL/LAP might partly be attributed to the effects on HUVECs. Previous studies had focused on the osteogenesis of BMSCs and the angiogenesis of endothelial cells after co-culture with LAP-containing biomaterials [36, 43, 44]. Page *et al.* found LAP would stimulate tubulogenesis of HUVECs *in vitro* and promote blood vessel growth *in vivo* [44]. Nevertheless, the present study provides new insight into evaluating the pro-regenerative properties of biomaterials by indirect co-culturing of BMSCs and endothelial cells.

Toxicological safety, biocompatibility and bioactivity are necessary in biomaterials designed for clinical oral-maxillofacial bone reconstruction applications [1]. The toxicological safety of the 3D-printed PCL/LAP scaffold was systematically evaluated after implantation into rat calvarial defects *in vivo*. Conventional body weight change and histological analyses of the liver, kidney and spleen were assessed, and no obvious abnormalities or substantial damage could be found. These findings are consistent with previous research focused on LAP-containing hydrogels that demonstrated toxicological safety [45]. Then, a 3D-printed porous PCL/LAP scaffold was customized for rat calvarial defect regeneration to further verify its effectiveness as a bone graft *in vivo.* After 12 weeks of implantation, a layer of new bone formation below the PCL scaffolds was observed. This result indicated that 3D-printed porous scaffolds with particular geometric shapes and structures could manipulate bone regeneration. Moreover, more mature and well-formed vascularized bone penetrated and filled the PCL/LAP scaffolds compared to the PCL scaffolds. Blood vessels were more plentiful within newly regenerated bone around the PCL/LAP scaffolds than around the PCL scaffolds. The newly formed bone around PCL/LAP scaffolds was more similar to natural bone, which is highly vascularized and maintains skeletal integrity through the vasculature for delivering oxygen, nutrients, hormones and growth factors. We speculated that these results might mostly contribute to the presence of LAP nanosilicates, which may be released from the PCL/LAP scaffold and taken up by cells, subsequently undergoing degradation within lysosomes [37, 46, 47]. The degradation products of LAP consist of silicon [Si(OH)_4_], magnesium (Mg^2+^) and lithium (Li^+^) ions [37, 48]. All these degradation products have been regarded as playing considerable roles in osteogenesis and angiogenesis [16, 37, 40]. These results confirm the osteoconductive and osteoinductive properties of LAP-incorporated PCL *in vivo* and suggest that LAP-incorporated PCL can be envisioned as a promising candidate for vascularized bone regeneration. Considering that bone is highly vascularized by nature and bone formation is always coupled with angiogenesis, a clear temporal influence on early-enhanced angiogenesis by LAP would be required in the future research.

Although the 3D-printed PCL/LAP scaffold showed excellent biocompatibility, biosafety and bioactivity in bone regeneration, there were some limitations of the scaffold in this study. No obvious degradation of the PCL/LAP scaffold occurred in the present study after 12 weeks of implantation. The long degradation period of the PCL/LAP scaffold might impede the new bone integration process *in situ*. Further modification of the PCL/LAP must focus on scaffold degradation. The particular mechanism of 3D-printed PCL/LAP scaffolds in BMSCs involved in bone regeneration is also worth further exploration.

## Conclusion

**Figure 9.**
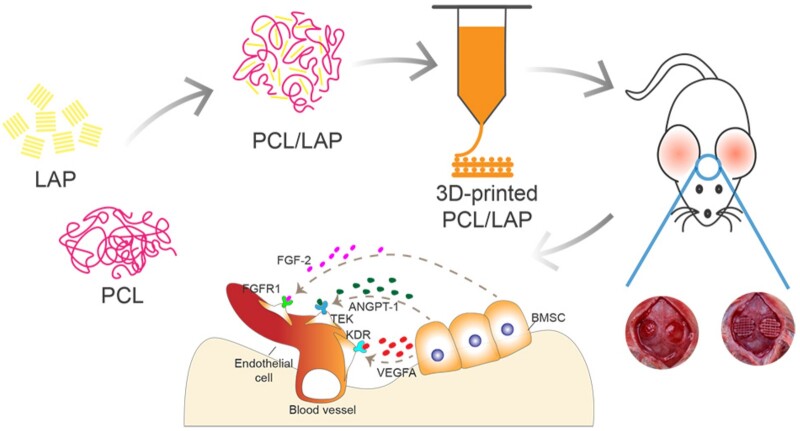
Schematic representation of the preparation of the PCL/LAP scaffold and its application in rat calvarial defect regeneration

In general, these results determined that LAP nanosilicate-functionalized PCL fabricated *via* 3D printing technology could augment vascularized bone regeneration to aid in the recovery of calvarial defects by orchestrating BMSCs (Fig. 9). The 3D-printed porous PCL/LAP scaffold enhanced cell viability, stimulated ALP activity, mineralized extracellular matrix formation and facilitated the osteogenic differentiation of BMSCs *in vitro*. Moreover, BMSCs cultured on PCL/LAP promoted angiogenesis potential by angiogenic cytokine secretion. The porous PCL/LAP scaffold has been proven to possess well biosafety, and its osteoconductive and osteoinductive properties provide a pro-regeneration microenvironment for vascularized bone regeneration of calvarial defects *in vivo*. We envision that the brilliant bioactivities of porous LAP nanosilicate-embedded PCL scaffolds fabricated by 3D printing technology would motivate their application in oral maxillofacial bone defect reconstruction.

## Funding

This study was supported by the National Natural Science Foundation of China (Grant No. 81870766), the Fujian Medical Innovation Project, Fujian Province (2020CXA048), the Fujian Medical Talents Training Project (2020GGA061) and the Startup Fund for scientific research, Fujian Medical University (Grant No. 2019QH2041).

*Conflict of interest statement*. The authors declare that they have no known competing financial interests or personal relationships that could have appeared to influence the work reported in this paper.
